# Achieving progress in maternal and neonatal health through integrated and comprehensive healthcare services – experiences from a programme in northern Tanzania

**DOI:** 10.1186/1475-9276-8-27

**Published:** 2009-07-30

**Authors:** Bjørg Evjen-Olsen, Øystein Evjen Olsen, Gunnar Kvåle

**Affiliations:** 1 Haydom Lutheran Hospital, Mbulu District, Manyara Region, Tanzania; 2 Centre for International Health, Overlege Danielsens House, Medical Faculty, University of Bergen, PO Box 7804, N-5020 Bergen, Norway; 3 DBL-Centre for Health Research and Development, University of Copenhagen, Copenhagen, Denmark

## Abstract

**Background:**

An integrated and comprehensive hospital/community based health programme is presented, aimed at reducing maternal and child mortality and morbidity. It is run as part of a general programme of health care at a rural hospital situated in northern Tanzania. The purpose was through using research and statistics from the programme area, to illustrate how a hospital-based programme with a vision of integrated healthcare may have contributed to the lower figures on mortality found in the area. Such an approach may be of interest to policy makers, in relation to the global strategy that is now developed in order to meet the MDGs 4 and 5.

**Programme setting:**

The hospital provides reproductive and child health services, PMTCT-plus, comprehensive emergency obstetric care, ambulance, radio and transport services, paediatric care, an HIV/AIDS programme, and a generalised healthcare service to a population of approximately 500 000.

**Programme description and outcomes:**

We describe these services and their potential contribution to the reduction of the maternal and neonatal mortality ratios in the study area. Several studies from this area have showed a lower maternal mortality and neonatal mortality ratio compared to other studies from Tanzania and the national estimates. Many donor-funded programmes focusing on maternal and child health are vertical in their framework. However, the hospital, being the dominant supplier of health services in its catchment area, has maintained a horizontal approach through a comprehensive care programme. The total cost of the comprehensive hospital programme described is 3.2 million USD per year, corresponding to 6.4 USD per capita.

**Conclusion:**

Considering the relatively low cost of a comprehensive hospital programme including outreach services and the lower mortality ratios found in the catchment area of the hospital, we argue that donor funds should be used for supporting horizontal programmes aimed at comprehensive healthcare services. Through a strengthening of the collaboration between government and voluntary agency facilities, with clinical, preventive and managerial capabilities of the health facilities, the programmes will have a more sustainable impact and will achieve greater progress in the reduction of maternal and neonatal mortality, as opposed to vertical and segregated programmes that currently are commonly adopted for averting maternal and child deaths. Thus, we conclude that horizontal and comprehensive services of the type described in this article should be considered as a prerequisite for sustainable health care delivery at all policy and decision-making levels of the local, national and international health care delivery pyramid.

## Background

Mortality and morbidity linked to pregnancy and childbirth have been drastically reduced in most high-income countries. Maternal mortality is one of the indicators showing the greatest disparity between high- and lower-income countries, with the proportion of maternal deaths in women of reproductive age being 0.7% in established market economies as opposed to 29.1% in Sub-Saharan Africa [1].

By being included as Millennium Development Goals (MDGs) 4 and 5, and in light of the newly formed Partnership for Newborn, Maternal & Child Health, maternal, perinatal and neonatal mortality have recently received added focus from the international policy makers.

In spite of increased international and national efforts, Tanzania has one of the highest maternal mortality ratios (MMR) in Sub-Saharan Africa, with national ratio estimates as high as 1100 in 1995 and 1500 in 2006 per 100 000 live births [2,3], whereas other studies have shown a range of MMR from 241 to 1099 per 100 000 live births [4-10].

Currently, there is a growing consensus among the scientific communities and policy makers that improved intrapartum care linked to the health centre and hospital levels may be the most effective strategy to reduce the burden of maternal deaths in areas where the burden is high. In its simplest form, this means increased skilled attendance at birth and improved emergency obstetrics services [11,12]. Apart from intrapartum care, antenatal and postpartum care also contributes to the continuum of care needed to reduce maternal deaths.

Parallel to these policy recommendations, there has also been an international debate on vertical versus integrated and comprehensive programmes. One of the current challenges facing low-resource settings is the fragmentation of various health interventions, including those related to maternal and perinatal health, into vertical components [11,13,14]. This may often be demanded by donors, who may need simple indicators and "quick-win" results. However, programmes exist in developing countries which focus on the integration of services with documented impact on maternal and child health. The effects of such programmes are often difficult to quantify scientifically, since formal evaluation, e.g. using epidemiological methods, is difficult. Therefore, description of programmes is important for the documentation of relevant experiences, although identification of direct cause-effect relationships in terms of reduced mortality as a result of such programmes may be difficult. In this article we present a wide-ranging healthcare programme in rural Tanzania that aims at maximising trust and confidence in the population by providing an integrated and comprehensive health service. The programme focuses both on prevention and on comprehensive care that seems to have resulted in an improvement to the long-term maternal and child health situation in the area.

## Programme setting

### Setting

The Haydom Lutheran Hospital (HLH) is a 400-bed hospital owned by the Mbulu Diocese of the Evangelical Lutheran Church in Tanzania, and is incorporated fully into the national health plan under the Ministry of Health. It was founded in 1955, is situated in the Mbulu District of the Manyara Region in northern and central Tanzania 300 kilometres from the nearest urban centre, and is an area with a predominantly poor, rural population.

The immediate catchment area of the hospital consists of four administrative divisions in three different districts in two regions. These are the Dongobesh and Haydom divisions in Mbulu District (Manyara region), the Basotu Division in Hanang District (Manyara Region) and the Nduguti Division in Iramba District (Singida Region).

Extrapolation from the National Census of 2002 shows how the immediate catchment area for HLH covered a population of approximately 286 000 in 2006, in a total of 74 villages or towns. The hospital as a first referral hospital covers a population of about 500 000, while the greater reference area covers a population of approximately 2 000 000 [15-18]. The area is ethnically and geographically diverse [19,20], with all four major language groups of sub-Saharan Africa represented, and situated at approximately 1700 metres above sea level on a highland plateau between two branches of the Great Rift Valley.

### Health facilities and collaboration

Each of the two regions neighbouring the hospital has a regional hospital and one district hospital per district, all government owned. None of these hospitals lie in the catchment area of the HLH, but all are collaborators with HLH within the national health plan. Within the catchment area for HLH there are two hospitals (being HLH and one additional relatively much smaller Lutheran hospital which is currently being rehabilitated), two health centres (both government), 19 government dispensaries and four dispensaries run by other religious voluntary agencies. The government and other religious facilities are financed by the government or their respective religious societies. HLH and its outreach programme are financed by donations from the Royal Norwegian Embassy in Tanzania, gifts, patient fees and government funding through staff and bed grants, and the Basket Fund.

The District Health Basket Fund is a fund initiated and funded by a group of international donors with the objective of strengthening the District Health Management Team's (DHMT) ability to provide services within a fixed set of interventions or activities. The donors provide a specific amount of funds per capita to the district (0.5 USD), and these are distributed according to a predetermined formula to dispensaries, health centres, hospitals and the District Medical Officer's administration in the district.

All the health facilities in the catchment area collaborate through the District Health Management Teams in the respective districts, with annual meetings. HLH is represented in the DHMT. Further, they assist each other through ambulance, radio and telephone contact, when there is shortage of vaccines, drugs or other supplies, and through the outreach programmes. The national programmes on tuberculosis, expanded programme of immunization (EPI), intermittent presumptive treatment (IPT) and insecticide treated nets (ITN) in the malaria programmes are all areas of close interaction.

In collaboration with the government authorities, coordinators for the various national public health programmes administrated through the regional and district health authorities, DHMTs, government and other voluntary agency facilities in the catchment area, HLH has developed and taken responsibility for an extensive outreach programme with Reproductive and child health services (RCHS) (including focused antenatal care, IPT, ITN, EPI and family planning), prevention of mother to child transmission of (PMTCT) of Human Immunodeficiency Virus (HIV), voluntary counselling and testing (VCT) of HIV and male mobile clinic (HIV and sexually transmitted disease (STD) testing, treatment and education) services. Funding in kind with testing kits and drugs for the national programmes are to be supplied by the government, but are often in shortage or out of stock.

For the RCHS programme there is one post with daily activities at the hospital and 27 outreach posts which are visited once a month. The outreach posts are not health facilities, but may be in a building available or under a tree in the respective villages. The VCT and male mobile clinic services from HLH cover all 74 villages in the catchment area on a rotating outreach basis. There are a total of 51 sites in the catchment area for activities directed towards PMTCT of HIV, where education as well as VCT is offered. HLH offers services to 44 of these (28 HLH-RCHS sites and 16 government or voluntary agency sites, mainly in the dispensaries). The remaining 7 sites are covered by the government (6) or other voluntary agencies (1). For the figures given in this article (table 1) we will refer to data collected through the HLH run programmes (for RCHS 28 sites, for PMTCT 44/51 sites), and not by government or other voluntary agency programmes (for PMTCT 7/51 sites), as these are not available in the format needed.

**Table 1:** Maternal and perinatal health, Haydom Lutheran Hospital with outreach RCHS and PMTCT clinics, Tanzania, 2006

***Indicators***	***Haydom Lutheran Hospital ***[29]
Inpatients	11082
Outpatients	50129
Health facility deliveries	3201
Home deliveries with nurse midwife, RCHS aides or traditional birth attendant	13
Pregnancy complications – in/outpatients	576
Caesarean sections and ruptured uterus operations	534 (525 + 9)
Maternal deaths – direct/indirect	19 (2/17)
Number of RCHS clinics (static/mobile)	1/27
Total women examined – RCHS	28113
Total children examined – RCHS	83007
PMTCT – women tested for HIV at RCHS clinics	5397
Estimated live births catchment area HLH [15,16]	11918
Percent estimated live births HIV tested in catchment area	45.3%
PMTCT – pregnant women tested	3884
PMTCT – lactating women tested	1513
HIV positive pregnant women	32
HIV positive lactating mothers	27
Uptake of HIV positive women in PMTCT-plus programme	42%
Cumulative number HIV positive women from RCHS/PMTCT included in care and treatment programme (Sep 2003–Dec 2006)	83
Cumulative number of HIV positive patients in the HAART care and treatment programme (Sep 2003–Dec 2006)	808
Deaths from HIV/AIDS	32
Total malaria cases (in/outpatients)	8876 (2481/6395)
Total malaria deaths (% deaths among all inpatients malaria cases)	156 (6.3%)
Total hospital deaths (% malaria deaths of all deaths)	752 (20.7%)
Total cerebral malaria cases	158
Total cerebral malaria deaths (% cerebral malaria deaths of all cerebral malaria cases)	51 (32.3%)
% Cerebral malaria deaths of total malaria deaths	20.7%

Sources: [15,16,29]

### Previous research on maternal health

According to the International Classification of Diseases (ICD-10) issued by the World Health Organization (WHO), a maternal death is defined as a death of a woman while pregnant or within 42 days of termination of pregnancy, irrespective of the duration and the site of the pregnancy, from any cause related to or aggravated by the pregnancy or its management, but not from accidental or incidental causes [21].

In order to compare indicators from the Haydom Lutheran Hospital with national indicators in table 2, we used the process indicators recommended by the WHO (tables 3 and 4) [22-24]. The figures from the Haydom area are based on studies done between 1995 and 2000.

**Table 2:** Antenatal and emergency obstetric care; comparison between study area and other estimates from Tanzania, 1995–2004

***Antenatal and obstetric care indicators ***[22]	***Estimates from programme catchment area and Mbulu district 1995–2000 ***[6,25,28-30,32]	***Study from other areas in Tanzania ***[4,5,7,8,26,27,33,34]	***National estimates WHO/DHS – Tanzania 1995–96 ***[2,9]	***National estimates WHO/DHS – Tanzania*** ***2000–2005 ***[3,10]
Maternal mortality ratio	382 (95% CI 250–560) [6]	241[4], 348–1099[5], 442[7],961[8]	1100 [2]	1500 [3]
Perinatal mortality ratio	27 (95% CI 22–33) [25]			42 [10]
Neonatal mortality ratio	17 (95% CI 13–21) [25]	58[7],68[27],82[26]	36.3 [9]	32 [10]
HIV infected pregnant women for PMTCT	42% (2006) [29]			6% [3]
Proportion of women attended at least once during pregnancy by trained personnel for reasons related to pregnancy	128% [28]		97% [9]	94% [10]
Proportion of all births in health facilities	57% [30]	47.5% and 37.3% [33]	46.5% [9]	47% [10]
Met need for basic and comprehensive EmOC	50.4% [30]	13.9% – 19.3% [34]		
Caesarean section as % of complications^a^	40.3% [32]			
Caesarean section rate	3.6% [32]	1.4% – 1.8% [33]	2.1% [9]	3.2% [10]
Case fatality rate^b ^(Comprehensive EMOC)	1.4% [32]	3.9% – 1.9% [33]		

a. The Caesarean section rate as percent of complications is the rate of Caesarean sections of the total number of obstetric complications needing intervention in a population.

b. The case fatality rate is estimated as the number of obstetric deaths from among all women with obstetric complications in EmOC facilities.

Sources: [2-10,22,25,28-30,32-34,27]

**Table 3:** Signal functions used to identify Basic and Comprehensive Emergency Obstetric Care

***Basic EmOC services*^1^**	***Comprehensive EmOC services*^2^**
1. Administer parenteral antibiotics	(1–6) All of those included in Basic EOC
2. Administer parenteral oxytocic drugs	7. Perform surgery (Caesarean section)
3. Administer parenteral anticonvulsants for pre-eclampsia and eclampsia	8. Perform blood transfusion
4. Perform manual removal of placenta	
5. Perform removal of retained products (e.g., manual vacuum aspiration)	
6. Perform assisted vaginal delivery	

^1^A basic EmOC facility is one that is performing all of functions 1–6.

^2 ^A comprehensive EmOC facility is one that is performing all of functions 1–8.

Source: [22]

**Table 4:** Process indicators and minimum acceptable levels for Safe Motherhood monitoring

*Key questions*	*Indicator*	*Minimum acceptable level*
**I. Coverage** 1. Is emergency obstetric care (EmOC) available and reasonably distributed?		
	A. Amount of emergency obstetric care (EmOC):	For every 500,000 population, there should be:
	Basic EmOC facilities	At least 4 Basic EmOC Facilities
	Comprehensive EmOC facilities	At least 1 Comprehensive EmOC facility
	B. Geographical distribution of EmOC Facilities	Minimum level for amount of EmOC services is met in sub-national areas
2. Are the women using maternity services at basic and comprehensive EmOC facilities?	C. Proportion of all births in Basic and comprehensive EmOC facilities	At least 15% of all births in the population take place in either Basic or Comprehensive EmOC facilities
3. Are the women who really need EmOC services using these services?	D. Met need for EmOC – Proportion of women estimated to have complications who are treated in EmOC facilities	At least 100% of women with obstetric complications (estimated as 15% of births) are treated in EmOC facilities
	E. Quantity of critical services Caesarean sections as a percentage of all Births	As a proportion of all births in the population, Caesarean sections account for not less than 5% nor more than 15%
**II. Performance** 4. Is treatment successful?		
	F. Quality of care Case fatality rate	The case fatality rate among women with obstetric complications in comprehensive EmOC facilities is less than 1%

Sources: Modified from [22-24]

**Figure 1:**
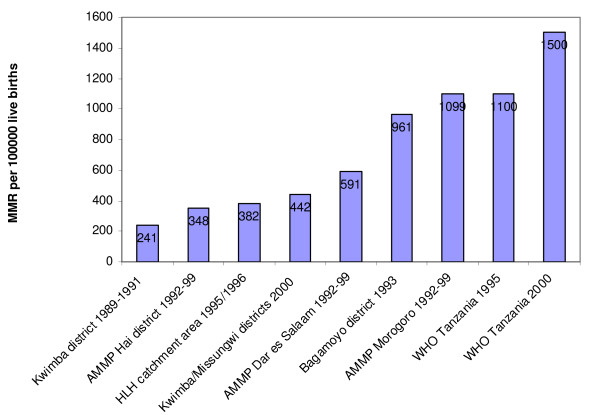
**Comparison of maternal mortality ratio estimates in Tanzania **[2-8].

**Figure 2:**
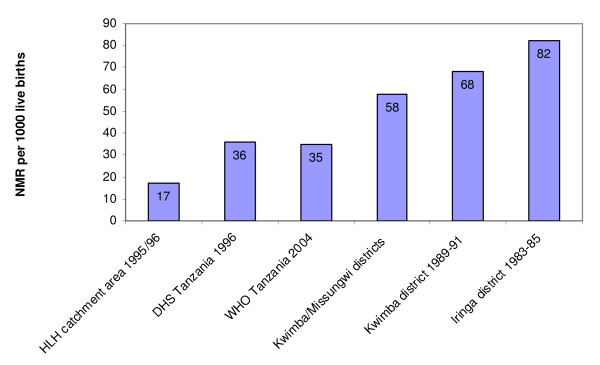
**Comparison of neonatal mortality ratio estimates in Tanzania **[3,7,9,25-27].

Previous research into the catchment area of the HLH programme showed an MMR of 382 per 100 000 live births (95%CI:250–560) in 1995–96, as compared to an MMR at the time of the study of 1100 per 100 000 live births on a national scale (figure 1) [2,3,6]. Furthermore, during the same time period of 1995–6, the studies found a perinatal mortality ratio (PMR) of 27 per 1000 births (95%CI:22–33) and a neonatal mortality ratio (NMR) of 17 per 1000 live births (95%CI:18–28) [25]. These figures are also lower than the national estimate of an NMR of 35 per 1000 live births and estimates from other studies (figure 2) [3,7,9,25-27].

## Programme description and outcomes

### Core hospital services

For five decades the hospital has focused on maintaining a wide range of services catering for the complex healthcare needs of the community. The priority-setting mechanisms have been guided by the demands of the communities, and the reality presented to the clinicians and households. The hospital has always tried to enable the clinicians to meet the multiple disease profiles presented by the community, as well as attempting to empower and enable the households, women, men and children to prevent diseases through educational and programmatic campaigns focusing on a very broad set of challenges.

In 2006 the hospital had three medical officers (MOs) in clinical service, five assistant medical officers (AMOs) and approximately 20 clinical officers. The MOs and AMOs perform Caesarean sections. The AMOs all are citizens of Tanzania, while the MOs are both Tanzanian and expatriate personnel. HLH has to rely on expatriate professional for some of the specialities, for the time being. However, many Tanzanian students from the Haydom area are now being trained as MOs, with the long-term plan being further specialisation. In addition, HLH recently became a field research site for the National Institute for Medical Research in Tanzania, resulting in an increasing number of Tanzanian masters and PhD candidates working in collaboration with the hospital.

### Emergency obstetric and postpartum services

The hospital qualifies as a Comprehensive Emergency Obstetric Care (CEmOC) facility and the health centres as Basic Emergency Obstetric Care (BEmOC) facilities (tables 3 and 4) [22]. At the hospital, nurse-midwives, RCHS aides and medical attendants staff the maternity ward, with AMOs and doctors covering the whole hospital, being available when called. In 2006 there were 3201 deliveries at HLH. A total of 534 Caesarean sections were performed. This percentage (16.7%) is hospital based. The WHO norm is 5–15% for a population. There are two main factors that are important in assessing Caesarean section rates. The first issue is hospital versus population based figures. Previous research in the catchment area of HLH showed that 56.9% of the women delivered at home and that the population based Caesarean section rate was 2.7% [6,28]. The women seek hospital care when complications arrive. Thus, the Caesarean section rate given above does not give a true picture of the population section rate.

The second issue is related to the level of skills of health personnel in performing assisted vaginal deliveries rather than Caesarean sections. The training in use of vacuum and forceps (operative vaginal deliveries) varies among doctors and midwives, both in Europe and Africa. Further, the nurse-midwife training in Tanzania has become more restrictive to the use of vacuum and forceps due to the HIV/AIDS situation. In addition, there may be a scarcity of senior obstetricians or doctors available to train junior doctors in these skills. At HLH the issue of training is currently being critically reviewed.

576 pregnancy complications were handled, with procedures that did not require Caesarean sections (table 1) [29].

Concerning the issue of skilled birth attendance, Olsen et al. [30] found 57% of births taking place in a health facility in the Mbulu and Hanang districts, which are both part of the HLH catchment area (table 2). There is a distinction between births taking place in a health facility and skilled birth attendance. We do not have exact figures giving this distinction, as one has to assess every birth in all health facilities in order to find this figure. At the HLH, every birth is attended to by a nurse-midwife, with 4 years of basic training at diploma level. Should complications arise, the doctor on call is summoned and appropriate action taken. The doctor on call is always required to stay on the hospital premises, carrying a mobile radio, in order to be rapidly available. In the case of emergencies, the basic theatre and anaesthetic staff are present at the hospital at all hours, and additional laboratory and theatre staff are collected at their homes by the hospital ambulance, should extensive surgery be required. All necessary intravenous fluids are produced at HLH.

The hospital has an extensive reproductive and child health service which covers large parts of the catchment area. Through this RCHS programme, women are advised to come to the hospital for delivery. Deliveries have been free of charge, but extra expenses such as operative procedures and medical treatment have not been free. However, the extra costs have been strongly subsidised by the hospital during the whole time scope of the programme, in order to make them affordable. The ambulance service has given first priority to delivering women on a 24-hour basis. From 2008, after having received grants from the Royal Norwegian Embassy for an MDG 4 and 5 programme, all ambulance services for delivering women and all costs related to delivery, including extra expenses, are free, in order to mobilise use of skilled attendance at birth in accordance with the aims of the MDGs 4 and 5. In addition, HLH has a nursing and midwifery school at diploma level. We believe these factors encouraging women to come for skilled attendance at birth may contribute to the lower perinatal, neonatal and maternal death rates found at HLH and in the HLH catchment area.

No patient needs to pay before service is given. The costs are calculated at discharge, and at that point in time personal financial constraints are taken into consideration.

There are no official figures on the use of traditional birth attendants (TBA) for the catchment area. The RCHS coordinator at the hospital has contact with the TBAs regularly in order to improve collaboration and referral to the hospital when complications arise. Previously, training of TBAs was a national policy, and HLH participated in this training. This has been abandoned as an international strategy, and thus also as a national strategy. Using the figures from Olsen et al.'s [30] study of 57% giving birth at a health facility, renders 43% giving birth outside a health facility, either at home or during transport. Presumably many of these will be attended to by a TBA. We also know that some deliver alone [28].

In 2006 there were 19 maternal deaths at the hospital. Of these, two were direct while 17 were indirect [29]. From an epidemiological study in the catchment area of the hospital in 1995/96, of 45 total maternal deaths, 29% (13 of 45) were direct obstetric deaths, while 71% (32 of 45) were indirect. Haemorrhage (38.5%) was the main cause of direct obstetric deaths (5 of 13). Cerebral malaria accounted for 44.4% of the total number of maternal deaths (20 of 45) and 62.5% of indirect obstetric deaths (20 of 32) [31]. From earlier studies the Caesarean section rate in the programme area was 3.6%, the met need for emergency obstetric care was 50.4%, and the proportion of women attending antenatal care at least once during pregnancy was 128% (most probably an influx of women from outside the catchment area) [28,30,32]. In table 2 these results are compared to other studies from Tanzania [9,10,30,33,34]. The operating theatre, intensive care unit and laboratory collaborate closely with the maternity ward, giving first priority to emergency obstetric conditions.

### Ambulance services

One unique feature of the emergency obstetric programme in this area is the ambulance service. The hospital has two 4-wheel drive vehicles, and in the 1990s approximately 200 kilometres of all-season feeder roads were built out to the villages. Each village has a contract with the hospital to maintain the roads, using the local community. The hospital will supply the machinery needed, while the villages supply the man power. If a village does not repair the roads, it will not receive ambulance or mother-and-child health services. Thus, the village leaders are under pressure from the community to maintain the roads. Each village also has a 24-hour solar-cell run Very High Frequency (VHF) radio placed in the homes of trusted families, with the hospital radio being in the reception where staff is available to respond on a 24-hour basis. Maternal cases are given first priority. In spite of heavy rains and at times nearly impassable roads, the ambulances are almost continuously on the road.

### Reproductive and child health services

The programme follows national guidelines using the "Focused Antenatal Care" package recommended by the World Health Organization as standard, as well as the standard vaccination and assessment guidelines for children [35]. The women are given free prophylaxis and treatment for anaemia and malaria. Furthermore, subsidised insecticide-treated bed-nets are provided for sale at a low cost at all sites, also following the national guidelines. In addition, the attendees are offered a full "prevention of mother-to-child transmission of HIV" (PMTCT-plus) package, which is integrated into the RCHS clinics (see below). From 2007, a syndromic management approach for sexually transmitted infections, with free treatment, was introduced at the RCHS sites. The antenatal attendees are advised to come to the hospital or health centres for delivery, and are familiarised with the healthcare setting through encounters with healthcare staff. The antenatal care clinics also provide information and education to mothers, men and children on a variety of preventive issues through a structured lecture programme at each clinic site.

A total of 28113 mothers and 83007 children were registered in 2006 from all 28 HLH-run RCHS clinics in the catchment area (table 1) [29].

### Prevention of mother-to-child transmission of HIV

HIV positive women, both pregnant and nursing, who have been tested at one of the 51 PMTCT sites mentioned earlier, are offered highly active antiretroviral therapy (HAART) and treatment for opportunistic infections. A follow-up system involving the RCHS, the PMTCT registry, the maternity ward, the care and treatment centre (CTC) for HIV/AIDS and the community home-based care counsellors (CHBCs) has recently been introduced in order to increase the uptake and adherence of both pregnant and lactating mothers to the HAART programme. In 2007, in the maternity ward, the hospital introduced provider-initiated counselling and testing with the opt-out strategy to all delivering mothers, in order to include more HIV positive mothers in the PMTCT programme.

The child is tested for HIV at 6, 12 and 18 months. The women and children are monitored with hospital and home visits by both the staff at the CTC and allocated CHBCs throughout pregnancy, lactation and until 18 months after delivery. Mothers are counselled on infant feeding options. Exclusive breastfeeding is encouraged more than replacement feeding, and mothers are provided with free food and milk aid according to their needs. In order to succeed with abrupt cessation of breastfeeding, the CTC and CHBC staffs follow up the mother closely around the date set for cessation. Social, nutritional and milk support is offered in order to ensure a safe transition. Those living close to the hospital receive milk from the HLH dairy cows, and those living further away are given financial support in order to ensure milk for the baby.

The mothers are given HAART until certain weaning has taken place, and should the staff be in doubt this is extended until 18 months after delivery when the child is tested the final time. Should the mother need HAART further for her own sake, its provision is continued.

In 2005, in the catchment area, 12 749 women were tested in the PMTCT/RCHS clinic sites. Of these, 52 women were found to be HIV positive, and 26 (50%) of these were enrolled in the HAART programme [36]. In 2006, there was a temporary drop in testing and uptake (42% uptake into PMTCT programme) due to the reorganisation from a vertical to an integrated HIV programme, but from 2007 the integrated programme was in place (table 1) [29]. This will hopefully lead to an increased rate of testing and uptake into the PMTCT programme. A national study in 2003–2004 showed an HIV prevalence of 2% in the Manyara Region and 3.2% in the Singida Region [37]. A previous study in the catchment area of the hospital showed an HIV prevalence of 2.0% (95%CI:1.34–2.97) among antenatal attendees [38].

### Integration of services

During the last two years of programme implementation the HIV services have been integrated from a vertical programme into normal hospital activities, largely through the RCHS, maternity and medical departments. This involves assigning the personnel regular working hours and salaries instead of using an allowance system. The allowance system paid per service rendered or per day of outreach or other type of work was more expensive than a system of regulated salaries. Furthermore, transport and activity costs and routines are planned in conjunction with the rest of the services provided. More importantly, the integration secures unified planning and reporting using the same internal control system. It also ensures a coordinated use, remuneration, motivation and training of staff, a unified organisational culture and improved interaction and synergies between departments and within the management structure. Currently PMTCT, VCT, RCHS and CTC services are coordinated with regards to all these aspects of quality and management issues.

### Costs and Payment

The total programme costs for the hospital, including all preventive and clinical care services and training, amount to approximately 3.2 million United States Dollars (USD) per year. Only 15–20 percent of the hospital income comes from patient fees, which are heavily subsidised at the outset. Approximately 6–7% of the budget is financed by grants from the Tanzanian government, and the remaining funds are covered by donors (mainly the Norwegian government through the Royal Norwegian Embassy) and gifts. One of the main contributors to poor patients being able to use the hospital is the fact that no payment is required for any service until discharge. This includes clinical as well as ambulance services, thus ensuring rapid help for the patients. If the patient has problems paying at discharge, the social worker at the hospital contacts village leaders in the patient's home village requesting information on the patient's economic situation. If they confirm the patient's situation, the debt is written off as exempted debt. In this manner, no matter what their income, all patients receive medical care. The exempted debt varies from year to year (from 8% to 20% of total cost-sharing income) according to harvest and rain conditions.

### Educational aspects

Education is an important aspect of improvement of maternal and perinatal health [39]. In the Manyara region, figures from the Demographic and Health Survey (DHS) from 2004 for women and men's education show that 31.8% of women and 9.4% of men had no primary school. 49.3% of women and 51.7% of men completed primary school. 3.8% of women and 3.4% of men had secondary school and the median number of years of schooling for both women and men was 6.1 years. For Singida region the figures were similar, with median years of schooling being 6.1 for women and 6.2 for men [10]. In the HLH catchment area there were 22 secondary schools in 2007.

In a recent study published on risk factors for maternal death from the HLH catchment area, 70% of both women and men in the study had between 1–7 years of formal education [40]. In this study, we found that the woman's educational level was not significantly associated with increased risk of maternal death. However, women with husbands who had no education had a significantly increased risk of maternal death. This may be an indication of the culture of the decision-maker's influence on whether the woman is allowed to seek obstetric care or not. The aspects of illiteracy and education in relation to maternal complications are discussed in detail in the study referred.

The HLH has always recognised how important education is for the survival of the mothers and their babies. Thus, from the early 1960's until the present time, the hospital has contributed substantially to building of primary and secondary schools in the whole wider catchment area. A secondary upgrading school functioned for approximately 10 years in the 1990's in order to assist staff and students to pass the national form four exams. Further, since 1983 the hospital has had a nursing and midwifery school, first at certificate level, and since upgraded into diploma level, producing 20 new nurse-midwives per year. HLH is currently planning to build a vocational training school for the area. Apart from formal education, HLH has since the early 1970's had an extensive RCHS work, with an outreach programme covering remote rural villages in a range up to 100 kilometres from the hospital. Every session has an hour's education on health related issues for the women attending, as described earlier. From 2003, 74 villages are reached by the HIV preventive work, where one offers VCT and educational sessions on HIV, sexually transmitted diseases (STDs) and general pregnancy, delivery and health related issues.

## Discussion

This article describes a comprehensive programme of integrated preventive and clinical care services related to maternal and child health outcomes in a low-income, rural setting. We wished to use all available research and statistical reports to illustrate the HLH programme and its focus on maternal and neo/perinatal health over several decades in relation to the current focus on MDG 4 and 5. Further, our intent was to illustrate how the HLH programme has had a vision of integrated healthcare that may have contributed to the lower figures on mortality found in this area. We think that this integrated approach may be of interest to policy makers, in that HLH has done in practice over many decades what is now being set forward as an acknowledged global strategy in order to meet the MDG 4 and 5 goals.

These outcomes are particularly related to the trust of the population. The estimates from the programme area show low levels of maternal and neonatal mortality compared to those from national figures provided by national estimates from Tanzania and other studies (figures 1 and 2 and table 2) [2-10,25-27].

Furthermore, table 2 compares process indicators related to antenatal and emergency obstetric care with other studies and national estimates [9,10,30,33,34]. Over the past years, the acceptance of the need to focus on the whole health system has been increasingly recognised [11,13,41]. It is therefore not surprising that a focus on the wide range of services needed and demanded in a population would lead to increased uptake and trust in the maternal and child healthcare services and thus also to a reduction in mortality levels.

### Challenges for comprehensive programmes

#### Collaboration within the national health plan

As described in the programme setting section, the HLH has an extensive collaboration with the regional and district health authorities and the other government and voluntary agency run health facilities in the catchment area., especially on the outreach programmes mentioned earlier. A greater understanding of the needs and level of care of each facility has been reached. This extensive collaboration, in the authors' view, may contribute to the positive developments seen in this area in relation to maternal and perinatal health.

Although the HLH is represented in the DHMTs, it has been difficult to gain equitable access to the ten percent of the Basket fund due to several reasons. In addition to the fact that district authorities have interpreted the policies such that voluntary agencies have only received the ten per cent of the funds remaining as "unallocated" in the guidelines, the fund covers all government and voluntary agency facilities in the district, thus allocating funds regardless of the extent of services rendered. Thus, a small dispensary may be allocated the same as the hospital, which covers a much broader range of services. In addition, the HLH catchment area covers three districts, with services such as RCHS, VCT, IEC, PMTCT, ITN, IPT, and EPI, including patients from a wider area. In addition, patients from all these districts come to the hospital for curative services. The HLH only receives basket funding from the Mbulu District (in terms of funds), where it is located geographically, and Hanang District (in kind as medical supplies), even though the health and government authorities acknowledge and politically appreciate the hospital and outreach services covering all the 7 neighbouring districts.

The hospital is, as all the other health facilities in the area, also affected by the potential weaknesses of the system on the national level. Funding from the Ministry of Health through the established funding mechanisms (bed and staff grants) and allocation of trained staff is very low. Further, there is a constant shortage of testing kits and drugs for HIV, tuberculosis, sexually transmitted diseases, the diseases to be tested and treated in the focused antenatal care, IPT, ITN and vaccine programmes. All RCHS and PMTCT related services are free of charge following national pricing guidelines. The hospital is required to cover these shortages of drugs and equipment through its own funding mechanisms, thus providing these free services to the population through funds generated elsewhere.

#### Quality versus quantity

There are some important characteristics and challenges common to the provision of comprehensive services. The services need to attain a minimum level of quality. In a previously published article it has been discussed and shown that it is important to focus on critical levels of quality rather than on quantity of services [42]. Given the extreme lack of resources, this is an important distinction. Qualified personnel needed to reduce maternal and neonatal child mortality can logically only be placed at higher levels of services to adequately utilise the resources available at these facilities. These are resources such as operating theatre facilities, intensive care units and laboratories. It follows therefore that in order to reduce maternal and child mortality in the intrapartum and postpartum phases of the delivery without adding resources, the mothers need to be able to access facilities with these quality levels, which often forces them to travel long distances and bypass low quality services [43,44]. It is vital that policies do not encourage an increased quantity of services without ensuring the availability of a minimum level of quality and human resources, such as for instance that described in the EmOC framework [22,30,32,42].

#### Dichotomies in approaches to improving maternal and child health

Historically, there have been some dichotomies which have contributed to unintended conflicts of interest. Donor and government agencies, as well as national governments, have at times ended up setting maternal health against child health, short-term vertical programmes against long-term commitments with integrated programmes, community care against clinical care [13] and government versus voluntary agency facilities. These dichotomies may have slowed the process of obtaining an integrated, well-functioning health system, and may have drawn staff and funding away from the general healthcare services. The Haydom programme has been aware of these dichotomies for many years and has thus set out to provide a continuum of care: from antenatal care, to more recently the PMTCT-plus (aimed at mothers, fathers and their offspring), to transport and ambulance services, and finally to the delivery, postpartum and early childhood stages. All aspects are integrated into the regular health services. HLH has also brought the dichotomy between government and voluntary agency facilities up for discussion on higher policy levels for many years, both on an organisational level through the Christian Social Services Commission (CSSC) (an umbrella organisation for all church run health facilities in Tanzania), and in regular discussions with political and government authorities on all levels. In practice HLH has attempted to follow this through in its comprehensive health programmes.

By ensuring a continuum of care, and integrating preventive and clinical care services with the general health services, we believe trust will develop between the communities and the healthcare services. The issue of trust may be illustrated by the influx of women from outside the catchment area into the antenatal clinics (ANC) supervised by HLH, giving an ANC attendance of 128% in 1996 (table 2) [28]. Women will more likely become aware of the possibilities of life-saving interventions such as the prevention and treatment of malaria and HIV in pregnancy. Knowing that the main cause of indirect obstetric deaths both in this area and in another study from Uganda was malaria, and that without testing and treatment of HIV both the mother and baby are at risk of dying, integrating services may increase the women's use of healthcare services if complications should arise in pregnancy or postpartum [31,45]. We believe trust is created by both the integration of services, a continuum of care, and quality general healthcare services provided as a foundation for more specific programmes aimed at mothers and children.

#### Vertical versus integrated approaches

Another challenge to the continuation of this programme during the last decade has been the verticalisation of interventions introduced. The hospital services were in some areas fragmented into vertical programmes with separate funding and administration policies, such as the HIV/AIDS programme. During the three years of implementation of the HIV/AIDS services of the hospital, it was realised that the vertical nature of the programme was seriously detrimental to the overall services of the hospital. The main reason for this was the funding structure by the donors, since funds were linked to specific programme components based on the need of the donors to justify their involvement. In addition, the national implementation guidelines provided incentive and allowance structures different from those offered to other health services and personnel, generating a strong incentive for the most qualified and leadership personnel to "defect" to the HIV/AIDS programme and thus rendering the rest of the hospital at times without qualified personnel when needed. It was clear to both the donors and the administration of the hospital that there was a need for change. The integration process, although painful to the staff involved because of reduced income, was necessary and inevitable.

#### Costs and funding

There are major challenges to achieving a broader acceptance as well as to implementing this described comprehensive programme. One major issue is the low level of funds available to comprehensive programmes. It is often believed that comprehensive programmes are expensive and not cost-effective. The description of the programme setting shows that the HLH is the dominant provider of services in its catchment area, enabling further analysis of programme costs per catchment area capita. This programme shows that at a total cost of 3.2 million USD as many as 500 000 people are secured a wide range of health services, both preventive and care-oriented. At a cost of USD 6.4 per capita this should be considered feasible for many low-income countries. Although this figure does not attempt to provide a cost-effectiveness measure in terms of disability adjusted life years (DALYs) or the equivalent, and can therefore not be used as a measure against the recommended USD 50 per DALY for approval of an intervention, it could usefully be compared with larger healthcare costs where countries such as Tanzania have made available USD 7 of government expenditure on health per capita [46]. We would therefore argue that it is likely that a comprehensive approach to population health is a cost-effective way of approaching health service delivery. It is certainly more feasible if, in addition to cost-effectiveness, we factor in all other values that are important to priority-setting in terms of the demands and needs of the community. These include the right to health care, care to marginalised groups, and the rule of rescue (i.e. treating people that are sick, regardless of the cost-effectiveness or efficacy of the intervention needed). These are values that are important to a community, and that contribute to trust in the health systems [47,48].

### The value of these findings for other settings

In this programme, the focus has been on guaranteeing a long-term commitment to both maternal and perinatal/neonatal care through well-functioning comprehensive general care with emergency obstetric care services provided at a hospital, with a close collaboration with all lower level health facilities (both government and other voluntary agencies (VAs)) in the area, while at the same time ensuring well organised preventive services within both antenatal care and PMTCT-plus. We believe that a holistic approach is necessary to establish a sense of trust in the population. The programme is in a rural area of Tanzania, where the majority of the population is poor. This type of setting is common to many areas of Africa. We think that this approach would be feasible in many settings similar to this area. However, this holistic approach needs the commitment of governments and donors to facilitate the long-term strategic planning involved. The importance of this comprehensive approach needs to be emphasised at both the planning and the funding levels. Although intuitively correct for both patient and provider, it is not always easy to convince planners and funding agencies. It is a greater challenge to fund and secure a comprehensive health programme such as that described here than to fund and implement a simpler vertical programme with a limited scope and time-span. However, the tendency to simplify and focus is not conducive to securing the trust and commitment to the programme from the beneficiaries [48]. Further, the authors believe that diversion of funding from vertical programmes to more integrated programmes could also cover the funding needed to ensure low cost or free delivery and child health care services.

### Comprehensive care and global policies

A final challenge worth discussing is the need for a global and national acceptance for the concepts related to integrated and comprehensive healthcare services in the quest to reduce maternal and child mortality and morbidity. Reich and colleagues discuss how donors have focused on disease-specific programmes and performance measures, leading to a fragmented array of uncoordinated programmes. These programmes have depended on a functioning health system as a foundation on which to build their programmes, often without supporting the underlying health systems. Thus, these programmes have at times not fully dealt with the broader system failures. These failures may lie behind the inadequate progress of many countries on key targets such as MDGs 4, 5 and 6 [49].

Although there is an increased focus on the effect of fragmented services on health systems, there is still insufficient emphasis on the need to provide comprehensive services at a minimum quality level to populations in low-income countries. Unless this becomes the main objective of global and national policies, as it is in most high- and middle-income countries, there is little reason to believe that a sustained reduction of maternal and child mortality can be achieved. There are many important value questions relevant to this concept, of which the basic right to health services is an important one. Mothers are sensitive to these values, and may not be persuaded into a sustained use of pregnancy and delivery services if their wider needs for healthcare are not also met.

## Conclusion

In summary, we have reported from a wide-ranging, comprehensive and integrated intervention programme aimed at providing for as many as possible of the most pressing healthcare needs and demands of a predominantly poor population in a rural setting in a low-income country. We believe the relatively low rates of maternal, perinatal and neonatal deaths in the area may be attributed to long-term access to comprehensive health services. Further, we believe a close collaboration between government and voluntary agency facilities, especially in outreach programmes aimed at maternal and perinatal health, is essential in order to cover rural areas with adequate services.

Such a programme increases trust and legitimacy, and thus also improves the effects of the maternal and child health interventions. We have learned some lessons from our setting. On a national and international level, the solution from our point of view could be to coordinate donor and national funding, training, implementation and monitoring issues to support comprehensive care through all health facilities in rural and semi-rural areas, rather than creating a division between government and voluntary agency facilities. The priority setting mechanisms must be based on the values of the beneficiaries to secure trust, with an integrated approach. This means ensuring that the facilities and care providers have a unified internal control system (planning, implementation, monitoring cycle), quality assurance procedures, remuneration and human resource policies that contribute to a productive and motivating organisational culture across all facilities. This should be considered as a prerequisite for sustainable health care delivery and coordination must be done at all levels of the local, national and international health care pyramid. The programme described may hopefully provide helpful insight to public health experts and policy makers.

## Abbreviations

AIDS: Acquired Immuno Deficiency Syndrome; AMO: Assistant Medical Officer; ANC: Antenatal Clinic; BEmOC: Basic Emergency Obstetric Care; CEmOC: Comprehensive Emergency Obstetric Care; CHBC: Community Home Based Care Counsellor; CO: Clinical Officer; CSSC: Christian Social Services Commission; CTC: Care and Treatment Centre; DALY: Disability Adjusted Life Year; DHMT: District Health Management Team; DHS: Demographic Health Survey; EmOC: Emergency Obstetric Care; EPI: Expanded Programme of Immunization; HAART: Highly Active Antiretroviral Therapy; HIV: Human Immunodeficiency Virus; HLH: Haydom Lutheran Hospital; ICD-10: International Classification of Diseases, version 10; IPT: Intermittent Presumptive Treatment; ITN: Insecticide Treated Nets; MDG: Millennium Development Goals; MMR: Maternal Mortality Ratio; MO: Medical Officer; NMR: Neonatal Mortality Ratio; PMR: Perinatal Mortality Ratio; PMTCT: Prevention of Mother to Child Transmission (used in relation to HIV); RCHS: Reproductive and Child Health Services; STD: Sexually Transmitted Disease; TBA: Traditional Birth Attendant; USD: United States Dollars; VA: Voluntary Agency; VCT: Voluntary Counselling and Testing; VHF: Very High Frequency (pertaining to radio systems); WHO: World Health Organization.

## Competing interests

The authors declare that they have no competing interests.

## Authors' contributions

BE-O took the initiative to write the article, participated in the discussion on the concept and approach of the manuscript, the analysis of the available data, and the writing of the manuscript. ØEO participated in the initiative to write the article, the discussion on the concept and approach of the manuscript, the analysis of the available data, and the writing of the manuscript. GK participated in the initiative to write the article, the discussion on the concept and approach of the manuscript, and the writing of the manuscript. All authors read and approved the final manuscript.
